# The item/order account of word frequency effects: Evidence from serial order tests

**DOI:** 10.3758/s13421-021-01144-7

**Published:** 2021-03-30

**Authors:** Ian Neath, Philip T. Quinlan

**Affiliations:** 1 Department of Psychology, Memorial University of Newfoundland, St. John’s, NL A1B 3X9 Canada; 2 University of York, York, UK

**Keywords:** Memory, Recall, Short term memory, Word frequency effect, Item/order hypothesis

## Abstract

According to the item/order hypothesis, high-frequency words are processed more efficiently and therefore order information can be readily encoded. In contrast, low-frequency words are processed less efficiently and the focus on item-specific processing compromises order information. Most experiments testing this account use free recall, which has led to two problems: First, the role of order information is difficult to evaluate in free recall, and second, the data from free recall show all three possible patterns of results: memory for high-frequency words can be better than, the same as, or worse than that for low-frequency words. A series of experiments tested the item/order hypothesis using tests where the role of order information is less ambiguous. The item/order hypothesis predicts better performance for high- than low-frequency words when pure lists are used in both immediate serial recall (ISR) and serial reconstruction of order (SRO) tests. In contrast, when mixed (alternating) lists are used, it predicts better performance for low- than for high-frequency words with ISR tests, but equivalent performance with SRO tests. The experiments generally confirm these predictions, with the notable exception of a block order effect in SRO tasks: When a block of low-frequency lists preceded a block of high-frequency lists, a high-frequency advantage was observed but when a block of high-frequency lists preceded a block of low-frequency lists, no frequency effect was observed. A final experiment provides evidence that this block order effect is due to metacognitive factors.

The term “word frequency paradox” is usually understood to refer to the finding that whereas lists of high-frequency words are better recalled than lists of low-frequency words (e.g., Deese, 1960; Peters, 1936; Sumby, 1963), the opposite result is found when memory is tested via recognition: Memory is better for lists of low-frequency words than lists of high-frequency words (e.g., Gorman, 1961; McCormack & Swenson, 1972; Schulman & Lovelace, 1970). However, a further paradox occurs when the high- and low-frequency words occur in the same list. In recognition, the low-frequency advantage remains (e.g., Dorfman & Glanzer, 1988; Schulman, 1967; Shepard, 1967), but in free recall, all three possible patterns have been reported. Sometimes high-frequency words are better recalled than low-frequency words (e.g., Balota & Neely, 1980; Hicks, Marsh, & Cook, 2005; Smith, Glenberg, & Bjork, 1978), sometimes there is no difference in recall of high and low frequency (e.g., Ozubko & Joordens, 2007; Ward, Woodward, Stevens, & Stinson, 2003; Watkins, LeCompte, & Kim, 2000), and sometimes low-frequency words are better recalled than high-frequency words (e.g., DeLosh & McDaniel, 1996; Duncan, 1974; May & Tryk, 1970). The latter appears to be the most frequently observed pattern.

In this paper, we evaluate the explanation offered by the item/order hypothesis for frequency effects (DeLosh & McDaniel, 1996). The major difference from most previous work is that rather than using free recall, where the role of order information is at best ambiguous, we use both immediate serial recall and serial reconstruction of order tests because the role of order information is more clear.

One reason for focusing on the item/order hypothesis is because its explanatory power goes beyond just frequency effects. A number of theorists (e.g., Einstein & Hunt, 1980; Hunt & McDaniel, 1993) had noted the apparent paradox that sometimes there is a mnemonic advantage for distinctive items (e.g., von Restorff, 1933), whereas other times there is a mnemonic advantage for similar or related items (e.g., Crowder, 1979). The key insight of Hunt and colleagues was that rather than being a paradox, these types of results can be seen as a trade-off between item-specific and relational (or order) information. Serra and Nairne (1993) suggested that this insight could also explain different results observed when experimenters use within- versus between-list designs. For example, Slamecka and Katsaiti (1987) found a generation effect – better recall for items that are generated in some fashion as opposed to being merely read – when both types of items were in the same list but found no effect of generation when pure lists of generated and pure lists of read items were compared. According to Serra and Nairne, the reason is that pure and mixed lists differ in the amount of item and order information that is available. In pure lists, the act of generating an item enhances item information relative to just reading an item, but in free recall, the item advantage may not be seen because subjects typically use an order-based retrieval strategy. In a mixed list, in contrast, having two types of items disrupts order information, but this reduction in the usefulness of order information now promotes reliance on item information and the item advantage can emerge.

DeLosh and McDaniel (1996) suggested that similar reasoning could provide a general explanation for differences in memory performance as a function of using mixed versus pure lists with manipulations such as bizarre versus normal items, humorous versus common items, and detailed versus simple pictures. DeLosh and McDaniel focused, in particular, on word frequency. According to their account, high-frequency words are processed efficiently because they are common, and therefore order information can be readily encoded. In contrast, low-frequency words are processed less efficiently because they are uncommon and the focus on item-specific processing compromises the recovery and maintenance of order information. This simple proposal has been highly influential and has been incorporated into the development of a recent computational account of frequency effects in memory (Popov & Reder, 2020). Not only does it offer a good account of the relevant extant findings, it also links the explanation of the frequency effect to other areas of memory research.

The item/order account offers an explanation for the two “word frequency paradoxes” noted earlier. Low-frequency words are better recognized than high-frequency words because recognition benefits more from item than order information. This obtains with both pure and mixed lists. In order to apply the idea to free recall, DeLosh and McDaniel (1996) assumed that order information helps free recall. With this assumption, the item/order account predicts that for pure lists, high-frequency words will be better recalled than low-frequency words. For mixed lists, the key is the extent to which, as noted by Serra and Nairne (1993), the presence of two types of items disrupts order information. DeLosh and McDaniel reasoned that in mixed lists,

“the order encoding normally associated with one type of item will be modulated by the presence of the alternative item type. This is because serial-order information for an item in any given serial position is necessarily dependent on the degree to which serial-order information for neighboring items is intact. Thus, in a mixed list of common and unusual items, the order encoding associated with common items will be somewhat disrupted relative to pure lists, whereas the order encoding associated with unusual items will be somewhat enhanced relative to pure lists.” (p. 1137).

Based on this, the item/order account predicts that for mixed lists, low-frequency words will be better recalled than high-frequency words because “order information is essentially equal for common and unusual items” (p. 1137) and therefore the low-frequency words will be better recalled due to their advantage in item information.

This latter prediction has been difficult to evaluate because all three patterns have been observed. At least one reason for the contrasting pattern of results may be variation in the degree to which order information is involved with free recall. For example, shorter lists tend to be recalled in order more than longer lists, and output order can also vary with delay (Spurgeon, Ward, & Matthews, 2014). Note that variability in order information could also affect the predictions for pure lists.

Given this, we assessed the predictions of the item/order hypothesis for both pure and mixed lists of high- and low-frequency words using immediate serial recall and serial reconstruction of tests in which the role of order information is less variable. Both tasks begin the same procedurally: A short list of words is shown one at a time followed immediately by a test. For serial recall, the instructions are to type or write down the words in the same order they were presented. For serial reconstruction of order, the items are given to the subject, either in alphabetical order or in a new random order, and the instructions are to click on the words in the same order they were presented. Without sufficient order information, performance on both tasks will be extremely low even if memory for the items themselves is perfect. In contrast, performance on a free-recall task can be perfect even in the complete absence of order information.

The item/order hypothesis makes predictions for both pure and mixed lists for each test. For immediate serial recall, the predictions are the same as for free recall, given the assumption of an order-based retrieval strategy in free recall. There should be a high-frequency advantage for pure lists because of the enhanced order information of the common words relative to the less common words. In mixed lists, there should be a low-frequency advantage: Given roughly equal order information, there will be a net benefit for low-frequency words due to their enhanced item information.

For serial reconstruction of order, the predictions of the item/order hypothesis for pure lists are identical to those for immediate serial recall for similar reasons: The high-frequency words benefit due to their enhanced order information. For mixed lists, however, the prediction is different: The item/order hypothesis predicts equivalent performance. As with serial recall, the presence of both types of items within the list reduces order information for the high-frequency items and enhances order information for the low-frequency items, resulting in roughly equivalent order information. However, presenting the items offsets the advantage the low-frequency items usually enjoy by enhancing item information for the high-frequency items. The net result is approximately equal recall. Note also that for both tests and for pure lists, the item/order account predicts a word frequency effect in between-subjects designs, which by definition use only pure lists, and also in blocked designs, which again use only pure lists.

Table 1 summarizes these predictions, and also documents which predictions have been tested in the literature. When frequency is manipulated within subjects using pure lists that are presented in a random order, there is a high-frequency advantage for both immediate serial recall (e.g., Roodenrys, Hulme, Alban, Ellis, & Brown, 1994; Roodenrys & Quinlan, 2000; Watkins, 1977) and serial reconstruction of order (Quinlan, Roodenrys, & Miller, 2017). This confirms the prediction of the item/order hypothesis, although we could find only one study that tests the latter prediction. When list order is blocked rather than randomized (i.e., half the subjects receive all of the low-frequency lists first, then all of the high-frequency lists second, and the other half of the subjects receive the blocks of lists in the opposite order), there are no extant studies to assess the prediction.

**Table 1:** The word frequency effect as a function of whether lists are pure or mixed (alternating); whether frequency is manipulated between- or within-subjects; whether list order is randomized or blocked; and whether the test is immediate serial recall (ISR) or serial reconstruction of order (SRO)

List type	Pure lists						Mixed Lists	
Frequency Manipulation	Within Subjects				Between Subjects		Within Subjects	
List order	Randomized list order		Blocked list order		N/A		N/A	
Test type	ISR	SRO	ISR	SRO	ISR	SRO	ISR	SRO
Predicted	Hi > Lo	Hi > Lo	Hi > Lo	Hi > Lo	Hi > Lo	Hi > Lo	Lo > Hi	Hi = Lo
Observed	Hi > Lo	Hi > Lo	Hi > Lo	Hi > Lo^a^ Hi = Lo^b^	Hi > Lo	Hi > Lo	Hi = Lo	Hi = Lo
Study	Roodenrys and Quinlan (2000); Roodenrys et al. (1994); Watkins (1977)	Quinlan et al. (2017); Exp. 1	Exp. 4 Exp. 7	Exp. 5 Exp. 6 Exp. 7	Neath and Surprenant (2019); Saint-Aubin and Poirier (2005); Stuart & Hulme (2005)	Exp. 3	Caplan et al. (2015); Hulme et al. (2003); Morin et al. (2006);	Exp. 2

Also shown are the predictions of the item/order hypothesis, the observed pattern, and studies showing that pattern

a When the low block came first (Exps. 5 and 6) or when the test type was not known (Exp. 7)

b When the high block came first and the test type was known (Exps. 5 and 6)

When frequency is manipulated between subjects (and therefore mixed lists are not possible), there is a high-frequency advantage for immediate serial recall (e.g., Neath & Surprenant, 2019; Saint-Aubin & Poirier, 2005; Stuart & Hulme, 2000), again confirming a prediction, but there are no extant studies for serial reconstruction of order.

For mixed lists, recall of high- and low-frequency words is equal for immediate serial recall (e.g., Caplan, Madan, & Bedwell, 2015; Hulme, Stuart, Brown, & Morin, 2003; Morin, Poirier, Fortin, & Hulme, 2006), which disconfirms the prediction of better memory for low- than high-frequency words, but there are no extant studies for serial reconstruction of order.

The purpose of the experiments was to provide data to evaluate the predictions in Table 1 that have not yet been tested.

## Experiment 1

Experiment 1 was a replication of Experiment 2 of Quinlan et al. (2017), who found better recall of high- than low-frequency words with pure lists in a serial reconstruction of order task. The primary change was using an online rather than an undergraduate subject pool. Subjects saw pure lists of high- and low-frequency words, and the lists were randomly intermixed. The test was serial reconstruction of order. The item/order account predicts that performance should be better for high- than low-frequency words because of the better processing of order information.

### Materials and methods

#### Subjects

Twenty volunteers from ProlificAC were paid £8 per hour (pro-rated) for their participation. The following inclusion criteria were used for this and all subsequent studies: (1) native speaker of English, (2) approval rating of at least 90% on prior submissions at ProlificAC, and (3) age between 19 and 39 years. The mean age was 29.50 years (*SD* = 5.15, range 21–38); ten self-identified as female, and ten self-identified as male. The sample size was based on observing ${\eta}_p^2$ = 0.231 for the main effect of frequency in Experiment 2 of Quinlan et al. (2017). A sample of 20 would yield power greater than 0.90 to detect this sized effect (Faul, Erdfelder, Buchner, & Lang, 2009).

#### Stimuli

The stimuli were the same as those used by Quinlan et al. (2017). See Table 1 of Appendix A of that paper for details.

#### Design

There were two within-subjects conditions: frequency (lists of high- or low-frequency words) and serial position.

#### Procedure

After reading an informed consent form and agreeing to participate, the subjects were reminded of the instructions. A trial began when the subject clicked on a button labelled “Start next trial.” Six words randomly drawn without replacement from the appropriate pool (i.e., high or low frequency) were shown one at a time for 1 s in the center of the screen in 24-pt Helvetica. After the final word was shown, the subjects saw a message that asked them to click on appropriately labelled buttons to recreate the presentation order. The words from the list were shown in alphabetical order. The subjects were informed that they needed to click on the first word first, the second word second, and so on. Once a button was selected, it could not be chosen again, nor could the response be changed. Six responses were required. There were 30 trials. Half the trials had low- and half the trials had high-frequency words, and the order of these trials was randomly determined for each subject. Subjects could take a break at any time by refraining from clicking on the “Start next trial” button.

### Results and discussion

For all experiments, we analyzed the data using both frequentist and Bayesian analysis of variance using JASP (JASP Team, 2019). For the former, non-integer degrees of freedom indicate the Geenhouse-Geisser sphericity correction was applied. For the latter, a Bayes Factor (BF) is reported. BF_10_ between 3 and 20 indicates positive evidence for the alternate hypothesis (and therefore evidence against the null hypothesis); BF_10_ between 20 and 150 indicates strong evidence, and BF_10_ greater than 150 indicates very strong evidence (Kass & Raftery, 1995). BF_01_ indicates evidence for the null hypothesis using the same scale. Main-effect models were evaluated with respect to a random-effects error model, and interaction models were evaluated with respect to a main-effects model.

**Fig. 1:**
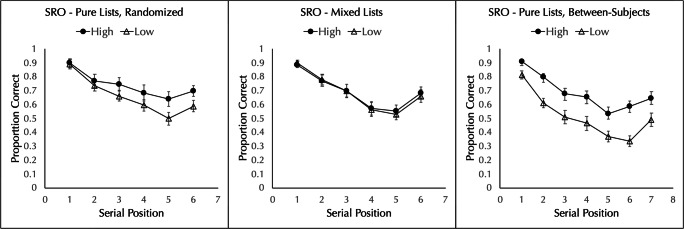
Proportion of high- and low-frequency words correctly placed in order in a strict serial reconstruction of order (SRO) test when pure lists are presented in random order (Experiment 1, left panel), mixed lists are presented (Experiment 2, middle panel), and pure lists are presented between-subjects (Experiment 3, right panel). Error bars show the standard error of the mean

The proportion of words correctly placed in order was analyzed by a 2 frequency (high vs. low) × 6 serial position repeated-measures ANOVA. As can be seen in the left panel of Fig. 1, there was a significant main effect of frequency, with better recall of high- (*M* = 0.739, *SD* = 0.167) than low- (*M* = 0.660, *SD* = 0.136) frequency words, *F*(1,19) = 10.300, *MSE* = 0.036, ${\eta}_p^2$ = 0.352, *p* = 0.005, BF_10_ = 71.49. The main effect of position was also significant, *F*(2.88,54.79) = 33.580, *MSE* = 0.027, ${\eta}_p^2$ = 0.639, *p* < 0.001, BF_10_ = 1.92×10^19^. The interaction was not significant, *F*(2.60,49.47) = 1.939, *MSE* = 0.024, ${\eta}_p^2$ = 0.093, *p* = 0.143, BF_01_ = 3.99.

Experiment 1 replicated the finding of Quinlan et al. (2017) that high-frequency words are better recalled than low-frequency words in a serial reconstruction of order task when pure lists are used and the order of the lists is random. This confirms a prediction of the item/order hypothesis and also demonstrates no meaningful difference between a university sample and an online sample.

## Experiment 2

Experiment 2 was identical to Experiment 1 except that mixed lists were used rather than pure lists. Half of the lists began with a high-frequency word and then alternated, and the other half began with a low-frequency word and then alternated. The item/order hypothesis predicts that recall of high- and low-frequency words will be equivalent because the presence of both types of items within the list reduces order information for the high-frequency items and enhances order information for the low-frequency items, resulting in roughly equivalent order information. In addition, presenting the items at test offsets the low-frequency advantage by enhancing item information for the high-frequency item.

*Subjects.* Twenty different volunteers from ProlificAC participated. The mean age was 27.60 years (*SD* = 6.34, range 20–39); 13 self-identified as female, and seven self-identified as male.

### Stimuli

The stimuli were the same as those used in Experiment 1.

### Design

Although Experiment 2 was again a 2 frequency (high vs. low) × 6 serial position repeated-measures design, each list contained both high- and low-frequency words.

### Procedure

The procedure was identical to that of Experiment 1 except for the following: Half the lists alternated beginning with a high-frequency word (i.e., HLHLHL), and the remaining lists alternated beginning with a low-frequency word (i.e., LHLHLH). The order of the lists was randomized for each subject.

### Results and discussion

Composite lists were created for data analysis. The first, third, and fifth words from the HLHLHL lists were combined with the second, fourth, and sixth words from the LHLHLH lists to construct composite high-frequency lists; the same was done to construct the composite low-frequency lists. The proportion of words correctly placed in order was analyzed by a 2 frequency (high vs. low) × 6 serial position repeated-measures ANOVA. Unlike in Experiment 1, and as can be seen in the middle panel of Fig. 1, there was no effect of frequency, with equivalent recall of high- (*M* = 0.694, *SD* = 0.152) and low- (*M* = 0.687, *SD* = 0.133) frequency words, *F*(1,19) = 0.494, *MSE* = 0.005, ${\eta}_p^2$ = 0.025, *p* = 0.491, BF_01_ = 6.90. Changing from pure lists to mixed lists abolished the frequency effect in serial reconstruction of order. The main effect of position was significant, *F*(3.08,58.60) = 37.551, *MSE* = 0.029, ${\eta}_p^2$ = 0.664, *p* < 0.001, BF_10_ = 8.22×10^31^. The interaction was not significant, *F*(3.20,60.76) = 0.209, *MSE* = 0.021, ${\eta}_p^2$ = 0.011, *p* = 0.900, BF_01_ = 33.20.

Experiment 2 found that alternating high- and low-frequency words within the same list abolishes the word-frequency effect in serial reconstruction of order, as predicted by the item/order hypothesis.

## Experiment 3

Experiment 3 also used serial reconstruction of order, but word frequency was now manipulated between subjects: Half the subjects received only high-frequency words and the other half received only low-frequency words. Two additional changes were that a new set of stimuli were used and the list length was seven rather than six.^1^ The item/order hypothesis predicts a word frequency effect will obtain because a between-subjects manipulation is just another type of pure list manipulation.

### Subjects

Forty different volunteers from ProlificAC participated. The mean age was 30.05 years (*SD* = 5.30, range 20–39); 24 self-identified as female, and 16 self-identified as male. The subjects were randomly assigned to one of the two groups.

### Stimuli

A set of 202 high- and 202 low-frequency words were created such that there was no overlap in frequency as measured by either CELEX (Medler & Binder, 2005) or SUBTLEX_US_ frequency (Brysbaert & New, 2009), and which were equated on the dimensions shown in the appendix. The high- and low-frequency words did differ in contextual diversity, but the two measures are highly correlated (i.e., *r* > 0.98 in the SUBTLEX_US_ corpus; see Guitard, Miller, Neath, & Roodenrys, 2019).

### Design

Word frequency was manipulated between subjects, and therefore only pure lists were used.

### Procedure

The procedure was similar to Experiments 1 and 2 except that subjects received 32 lists of only high-frequency words or 32 lists of only low-frequency words.

### Results and discussion

The proportion of words correctly placed in order was analyzed by a 2 word frequency (high vs. low) × 7 serial position mixed-factorial ANOVA. As can be seen in the right panel of Fig. 1, there was a main effect of frequency, with better performance for high frequency (*M* = 0.685, *SD* = 0.137) than low frequency (*M* = 0.512, *SD* = 0.144) lists, *F*(1,38) = 15.118, *MSE* = 0.139, ${\eta}_p^2$ = 0.285, *p* < 0.001, BF_10_ = 66.98. The main effect of position was significant, *F*(3.64,138.19) = 62.636, *MSE* = 0.022, ${\eta}_p^2$ = 0.622, *p* < 0.001, BF_10_ = 1.03 × 10^42^. The interaction was not significant, *F*(3.64,138.19) = 1.601, *MSE* = 0.022, ${\eta}_p^2$ = 0.040, *p* = 0.183, BF_01_ = 3.80.

As predicted by the item/order hypothesis, a word frequency effect obtains when frequency is manipulated between subjects and the test is serial reconstruction of order.

## Experiment 4

Experiment 4 used immediate serial recall and word frequency was manipulated within subjects but blocked: Half the subjects received a block of only high-frequency lists followed by a block of only low-frequency lists and the other half received the reverse order. The item/order hypothesis predicts a word frequency effect will obtain because the manipulation used pure lists.

*Subjects*. Forty different volunteers from ProlificAC participated. The mean age was 28.50 years (*SD* = 4.99, range 19–39); 22 self-identified as female, and 18 self-identified as male. The subjects were randomly assigned to one of the two groups.

### Stimuli

The stimuli were the same as in Experiment 3.

### Design

The order of the blocks of pure lists, high then low or low then high, was manipulated between subjects. There were 16 lists of each type.

### Procedure

The procedure was similar to Experiment 3 except that: (1) subjects saw a block of only high-frequency lists followed by a block of only low-frequency lists, or the reverse ordering, and (2) subjects were asked to type in their responses in strict serial order. If they could not remember a word, they were asked to either guess or click on a button labelled “skip”.

### Results and discussion

The proportion of words correctly placed in order was analyzed by a 2 block order (high then low or low then high) × 2 word frequency (high vs. low) × 7 positions mixed-factorial ANOVA. There was a main effect of frequency, with better performance for high-frequency (*M* = 0.612, *SD* = 0.185) than low-frequency (*M* = 0.416, *SD* = 0.157) lists, *F*(1,38) = 119.466, *MSE* = 0.038, ${\eta}_p^2$ = 0.759, *p* < 0.001, BF_10_ = 1.04 × 10^14^. The main effect of block order was not significant, *F*(1,38) = 1.265, *MSE* = 0.314, ${\eta}_p^2$ = 0.032, *p* = 0.268, BF_01_ = 2.82. The main effect of position was significant, *F*(2.31,87.91) = 95.163, *MSE* = 0.072, ${\eta}_p^2$ = 0.715, *p* < 0.001, BF_10_ = 6.07 × 10^61^.

**Fig. 2:**
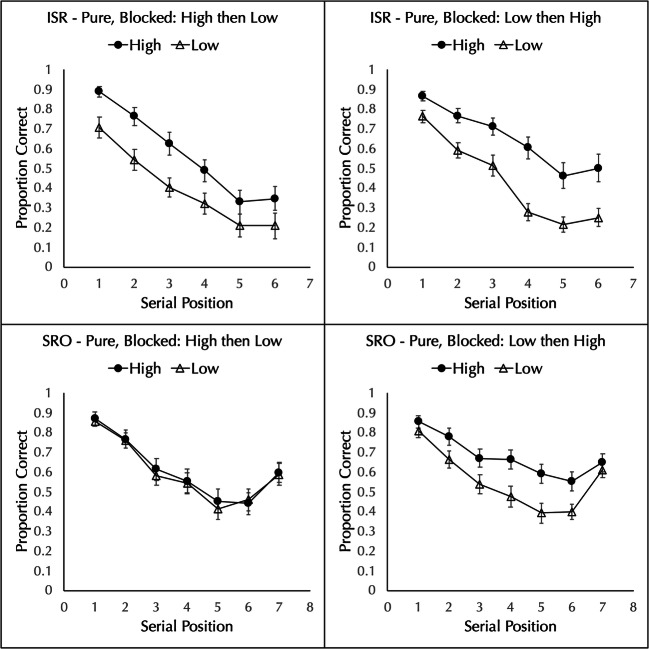
Proportion of high- and low-frequency words correctly recalled in a strict immediate serial recall (ISR) test in Experiment 4 (top row) and the proportion correctly placed in order in a serial reconstruction of order (SRO) test in Experiment 5 (bottom row) when pure lists are presented blocked. The left panels show a block of high-frequency lists followed by a block of low-frequency lists and the right panels shows the reverse. Error bars show the standard error of the mean

The interaction between block order and frequency was not significant, *F*(1,38) = 1.322, *MSE* = 0.028, ${\eta}_p^2$ = 0.034, *p* = 0.257, BF_01_ = 4.87. As can be seen in the top row of Fig. 2, there is a word frequency effect regardless of the order of the blocks.

Neither the frequency by position interaction, *F*(3.47,131.72) = 1.951, *MSE* = 0.017, ${\eta}_p^2$ = 0.049, *p* = 0.115, BF_01_ = 5.52, nor the position by block order interaction, *F*(2.31,87.91) = 0.751, *MSE* = 0.072, ${\eta}_p^2$ = 0.019, *p* = 0.493, BF_01_ = 38.01, were significant. The three-way interaction was significant by the frequentist test, *F*(3.47,131.72) = 4.360, *MSE* = 0.017, ${\eta}_p^2$ = 0.103, *p* = 0.004, but the Bayesian test was inconclusive, BF_10_ = 0.781.

As predicted by the item/order account, a frequency effect was observed in pure lists on an immediate serial recall test regardless of the block order.

## Experiment 5

Experiment 5 was identical to Experiment 4 except that that the test was serial reconstruction of order. The item/order hypothesis predicts a word frequency effect will obtain because the manipulation used pure lists.

### Subjects

Forty different volunteers from ProlificAC participated. The mean age was 29.80 years (*SD* = 5.86, range 19–39); 23 self-identified as female, and 17 self-identified as male. The subjects were randomly assigned to one of the two groups.

### Stimuli

The stimuli were the same as in Experiments 3 and 4.

### Design

The design was the same as Experiment 4.

### Procedure

The procedure was similar to Experiment 4 except for the test.

### Results and discussion

The proportion of words correctly placed in order was analyzed by a 2 block order (high then low or low then high) × 2 word frequency (high vs. low) × 7 positions mixed factorial ANOVA. There was a main effect of frequency, with better performance for high-frequency (*M* = 0.646, *SD* = 0.181) than low-frequency (*M* = 0.577, *SD* = 0.162) lists, *F*(1,38) = 22.598, *MSE* = 0.029, ${\eta}_p^2$ = 0.373, *p* < 0.001, BF_10_ = 701.60. The main effect of block order was not significant, *F*(1,38) = 0.040, *MSE* = 0.384, ${\eta}_p^2$ = 0.001, *p* = 0.842, BF_01_ = 3.55. The main effect of position was significant, *F*(3.51,133.24) = 78.189, *MSE* = 0.035, ${\eta}_p^2$ = 0.673, *p* < 0.001, BF_10_ = 2.98 × 10^69^.

However, there was a significant interaction between block order and frequency, *F*(1,38) = 14.674, *MSE* = 0.029, ${\eta}_p^2$ = 0.279, *p* < 0.001, BF_10_ = 41.77. As can be seen in the bottom row of Fig. 2, when the block of low-frequency lists occur first (right panel), performance is better for high-frequency lists (*M* = 0.679, *SD* = 0.159) than for low-frequency lists (*M* = 0.554, *SD* = 0.150), *t*(19) = 5.706, *d* = 1.277, *p* < 0.001, BF_10_ = 1375.6. In contrast, when the block of high-frequency lists occur first (left panel), performance with high-frequency lists (*M* = 0.613, *SD* = 0.200) is the same as in low-frequency lists (*M* = 0.600, *SD* = 0.175), *t*(19) = 0.700, *d* = 0.157, *p* = 0.492, BF_01_ = 3.46.

The frequency by position interaction was just significant by the frequentist test, *F*(4.84,183.87) = 2.318, *MSE* = 0.013, ${\eta}_p^2$ = 0.057, *p* = 0.047, but the Bayesian analysis did not offer support for this, BF_10_ = 0.806. Neither the position by block order (*F*(3.51,133.24) = 1.265, *MSE* = 0.035, ${\eta}_p^2$ = 0.032, *p* = 0.288, BF_01_ = 13.63) nor the three-way interaction (*F*(4.84,183.87) = 1.831, *MSE* = 0.013, ${\eta}_p^2$ = 0.046, *p* = 0.111, BF_01_ = 5.59) were significant.

The prediction of the item/order hypothesis is that a frequency effect would obtain when pure lists were blocked and the test was serial reconstruction of order. This prediction was supported only when the block of low-frequency lists came first; when the block of high-frequency lists came first, there was no frequency effect. Given the surprising result that block order affects whether a frequency effect will be observed, Experiment 6 was designed as a replication.

## Experiment 6

Experiment 6 used a free reconstruction of order test to see whether the block order effect was unique to serial reconstruction of order. A serial reconstruction of order test requires that the first word be chosen first, the second word chosen second, and so on. In contrast, a free reconstruction of order test allows any word to be the first response. In addition, set size was manipulated. Roodenrys and Quinlan (2000) have shown that set size can interact with frequency in some situations. The item/order hypothesis makes the same prediction for free reconstruction of order as for serial reconstruction of order for a blocked design.

### Subjects

Forty University of York undergraduates participated for course credit.^2^ The subjects were randomly assigned to one of the two groups.

### Stimuli

The stimuli were the same as in Quinlan et al. (2017) and Experiments 1 and 2. In the open set, there were 96 high- and 96 low-frequency words. In the closed set, six high- and six low-frequency words were drawn at random from the larger pool for each subject and were used on every trial.

### Design

The order of the pure lists, high then low or low then high, was manipulated between subjects.

### Procedure

There were 16 lists in each block, either all high-frequency or all low-frequency words. As in previous experiments, each word was shown for 1 s. At test, the words were shown in a single column on the left side of the display and the subject used the mouse to click on a word on the left and then click on a location on the right side of the list to indicate that item’s position.

### Results and discussion

The proportion of words correctly placed in order was analyzed by a 2 set size (open vs. closed) × 2 block order (high first vs. low first) × 2 frequency (high vs. low) × 6 serial position factorial ANOVA.

There was no effect of set size, with equivalent performance in the open (*M* = 0.776, *SD* = 0.137) and closed (*M* = 0.778, *SD* = 0.099) groups, *F*(1,76) = 0.009, *MSE* = 0.173, ${\eta}_p^2$ = 0.000, *p* = 0.923, BF_01_ = 3.58. Only one interaction involving set size was significant: frequency by set size by order, *F*(1,76) = 4.866, *MSE* = 0.034, ${\eta}_p^2$ = 0.060, *p* = 0.030, BF_10_ = 8.66. This reflects a larger difference in the magnitude of the frequency effect between the two orders in the closed condition (-0.036 for HiLo vs. 0.137 for LoHi) than in the open condition (0.013 for HiLo vs. 0.081 for LoHi). Because all other interactions involving set size were *F* < 1.00, *p* > 0.609, BF_01_ > 40, and to aid clarity, the data were collapsed over set size for the remaining analyses.

There was a significant main effect of frequency, with better performance for high- (*M* = 0.801, *SD* = 0.128) than low- (*M* = 0.753, *SD* = 0.139) frequency lists, *F*(1,78) = 16.083, *MSE* = 0.035, ${\eta}_p^2$ = 0.171, *p* < 0.001, BF_10_ = 94869. The main effect of block order was not significant, *F*(1,78) = 0.024, *MSE* = 0.170, ${\eta}_p^2$ = 0.000, *p* = 0.876, BF_01_ = 4.83. The main effect of position was significant, *F*(2.96,230.86) = 52.034, *MSE* = 0.026, ${\eta}_p^2$ = 0.400, *p* < 0.001, BF_10_ = 9.39 × 10^41^.

**Fig. 3:**
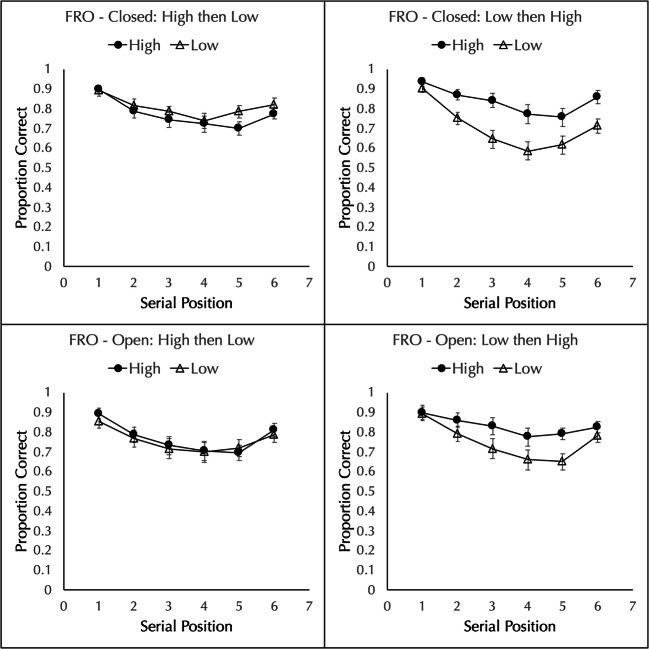
Proportion of high- and low-frequency words correctly recalled in an immediate free reconstruction of order (FRO) task in Experiment 6. The top row shows the results for a closed set and the bottom row shows the results for an open set. The left columns show a block of high-frequency lists followed by a block of low-frequency lists and the right panels shows the reverse. Error bars show the standard error of the mean

As in Experiment 5, there was a significant frequency × block order interaction, *F*(1,78) = 24.542, *MSE* = 0.035, ${\eta}_p^2$ = 0.239, *p* < 0.001, BF_10_ = 4.51 × 10^8^. As can be seen in Fig. 3, when the block of low-frequency lists occur first (right panel), performance is better for high-frequency lists (*M* = 0.834, *SD* = 0.125) than for low-frequency lists (*M* = 0.725, *SD* = 0.140), *t*(39) = 6.559, *d* = 1.037, *p* < 0.001, BF_10_ = 167625. However, when the block of high-frequency lists occur first (left panel), performance with high-frequency lists (*M* = 0.769, *SD* = 0.125) is the same as in low-frequency lists (*M* = 0.781, *SD* = 0.133), *t*(39) = 0.646, *d* = 0.102, *p* = 0.522, BF_01_ = 4.82.

Neither the frequency by position interaction, *F*(3.85,299.99) = 1.790, *MSE* = 0.011, ${\eta}_p^2$ = 0.022, *p* = 0.133, BF_01_ = 69.21, nor the position by block order interaction, *F*(2.96,230.86) = 1.198, *MSE* = 0.026, ${\eta}_p^2$ = 0.015, *p* = 0.311, BF_01_ = 49.70, were significant. The three-way interaction was significant, *F*(3.85,299.99) = 5.999, *MSE* = 0.011, ${\eta}_p^2$ = 0.071, *p* < 0.001, BF_10_ = 8.25. Inspection of this interaction suggests it is due to a larger difference between high- and low-frequency words in the closed pool condition when the low block precedes the high block than in the open pool condition, but no difference between the closed and open pools when the high block precedes the low block.

## Discussion of Experiments 5 and 6

Experiment 6 replicated the pattern observed in Experiment 5 despite numerous changes: A frequency effect was observed only when the low-frequency lists were tested first. Whatever caused the frequency effect to disappear in the high-low condition of a blocked design must be occurring after the completion of the first block because a standard frequency effect is observed in a between-subjects design, regardless of whether the test is immediate serial recall (Neath & Surprenant, 2019; Saint-Aubin & Poirier, 2005; Stuart & Hulme, 2000) or serial reconstruction of order (Exp. 3).

One possibility, then, is that on average, people think that immediate serial recall tests are more difficult than reconstruction of order tests because they have to produce the word, whereas for the latter, the words are provided. This leads to their (for want of a better expression) “trying harder” on the serial recall test than on the reconstruction of order test. Some evidence consistent with this comes from serial recognition test data. In this test, a short list of items is presented at study, and at test, the same items are again presented. On half the trials, two items are transposed in the second list and the task is to indicate if the items are in the same or in a different order as the first list. Chubala, Surprenant, Neath, and Quinlan (2018) found that performance on the serial recognition test was lower when it was the only test, but higher when half the trials could end in a serial recall test. There is other evidence for a metacognitive component for tasks involving frequency (e.g., Higham, Bruno, & Perfect, 2010; Tullis & Benjamin, 2012).

With an immediate serial recall test, the first experience is consistent with the expectation that the task is difficult regardless of the condition. In contrast, for reconstruction of order tests, the expectation can be consistent if the stimuli are “easy” and but can be less consistent if the stimuli are “hard.” The processing may change as a function of the extent to which the experience matches the expectation. It is more discrepant if the hard condition is first, and this is the condition in which the frequency effect was observed. It is less discrepant if the easy condition is first and this is the condition in which the frequency effect was not observed.

If this is the case, then the following prediction should hold. If the subjects do not know in advance whether they will receive an immediate serial recall or a serial reconstruction of order test, the block order effect should be eliminated. The reason is that the possibility of receiving an immediate serial recall test will cause the subjects to “try harder” on every trial. Experiment 7 was designed to test this prediction.

## Experiment 7

In Experiment 7, subjects did not know whether each list would be followed by an immediate serial recall or a serial reconstruction of order test until after list presentation. The predictions from the tentative metacognitive explanation are that: (1) the immediate serial recall trials should show no block order effect, as in Experiment 4, and (2) the reconstruction of order trials should also show no block order effect, the opposite result to that seen in Experiments 5 and 6. The reason is that because an immediate serial recall test is possible on each trial, the subjects “try harder” on every trial.

### Subjects

Fifty different volunteers from ProlificAC participated.^3^ The mean age was 28.60 years (*SD* = 5.77, range 20–39); 37 self-identified as female, and 13 self-identified as male. The subjects were randomly assigned to one of the two groups.

### Stimuli

The stimuli were the same as in Experiments 3–5.

### Design

The design was similar to Experiment 5, in that half of the subjects received a block of high-frequency lists first and the other half received a block of low-frequency lists first. It differed in that two types of test, immediate serial recall or serial reconstruction of order, were possible on every trial and the type of test was not known until after the list had been presented.

### Procedure

The procedure was similar to Experiment 5 except that subjects were informed that half of the trials would be followed by an immediate serial recall test and the other half of the trials would be followed by a serial reconstruction of order test. There were 40 lists in total, 20 in each block. Within each block, ten lists were followed by an immediate serial recall test and ten were followed by a serial reconstruction of order test. The order of the tests was randomized for each subject.

### Results and discussion

The immediate serial recall and serial reconstruction of order data were analyzed separately.

#### Immediate serial recall

The proportion of words correctly recalled in order was analyzed by a 2 block order (high then low or low then high) × 2 word frequency (high vs. low) × 7 positions mixed-factorial ANOVA. There was a main effect of frequency, with better performance for high-frequency (*M* = 0.527, *SD* = 0.206) than low-frequency (*M* = 0.374, *SD* = 0.195) lists, *F*(1,48) = 68.846, *MSE* = 0.060, ${\eta}_p^2$ = 0.589, *p* < 0.001, BF_10_ = 5.12 × 10^10^. The main effect of block order was not significant, *F*(1,48) = 0.127, *MSE* = 0.503, ${\eta}_p^2$ = 0.003, *p* = 0.723, BF_01_ = 3.55. The main effect of position was significant, *F*(2.56,122.69) = 115.626, *MSE* = 0.090, ${\eta}_p^2$ = 0.707, *p* < 0.001, BF_10_ = 4.35 × 10^96^.

**Fig. 4:**
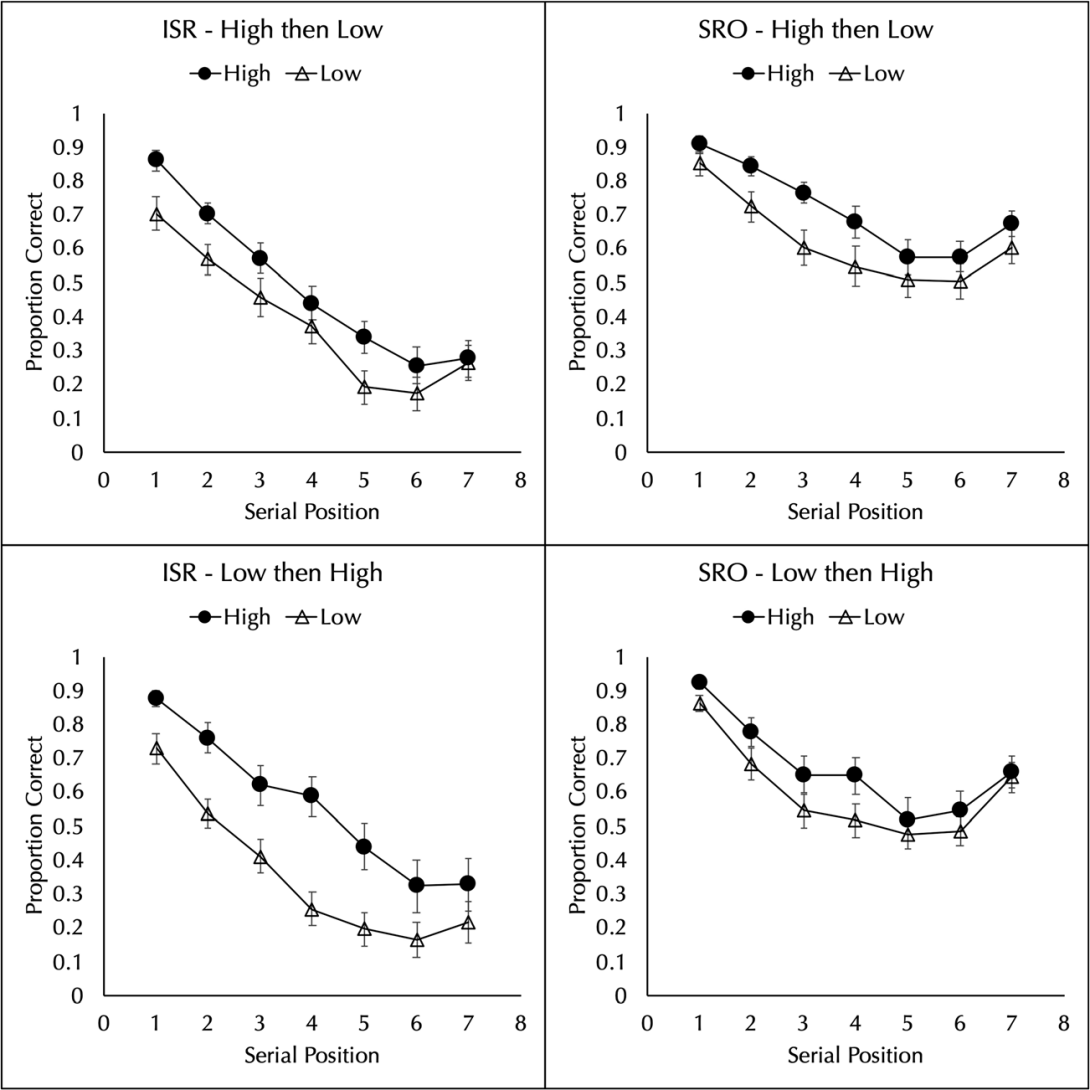
Proportion of high- and low-frequency words correctly recalled in an immediate serial recall (ISR) and serial reconstruction of order (SRO) test in Experiment 7 when the type of test was not known until after list presentation. The top row shows the results for the group that had a block of high-frequency lists followed by a block of low-frequency lists and the bottom row shows the results for a group that had the reverse. Error bars show the standard error of the mean

There was a significant interaction between block order and frequency, *F*(1,48) = 7.48, *MSE* = 0.060, ${\eta}_p^2$ = 0.135, *p* = 0.009, although the Bayes Factor offered little support, BF_10_ = 2.54. As can be seen in the left column of Fig. 4, to the extent that this interaction is real, the frequency effect is larger when the low frequency block occurs first than when the high frequency block occurs first. Nonetheless, there is a high-frequency advantage for each block order. When the block of low-frequency lists occur first, performance is better for high-frequency lists (*M* = 0.562, *SD* = 0.233) than for low-frequency lists (*M* = 0.359, *SD* = 0.206), *t*(24) = 8.629, *d* = 1.726, *p* < 0.001, BF_10_ = 1.58 × 10^6^. When the block of high-frequency lists occur first, performance is better for high-frequency lists (*M* = 0.493, *SD* = 0.172) than for low-frequency lists (*M* = 0.390, *SD* = 0.185), *t*(24) = 3.617, *d* = 0.723, *p* < 0.001, BF_10_ = 26.20.

The frequency by position interaction was significant, *F*(4.32,207.46) = 3.368, *MSE* = 0.024, ${\eta}_p^2$ = 0.066, *p* = 0.009, but again the Bayesian analysis contradicted this result, BF_01_ = 5.82. The position by block order interaction was not significant, *F*(2.56,122.69) = 0.201, *MSE* = 0.090, ${\eta}_p^2$ = 0.004, *p* = 0.867, BF_01_ = 264.44. The three-way interaction was not significant, *F*(4.32,207.46) = 2.348, *MSE* = 0.024, ${\eta}_p^2$ = 0.047, *p* = 0.051, BF_01_ = 73.01.

#### Serial reconstruction of order

The proportion of words correctly placed in order was analyzed by a 2 block order (high then low or low then high) × 2 word frequency (high vs. low) × 7 positions mixed-factorial ANOVA. There was a main effect of frequency, with better performance for high-frequency (*M* = 0.696, *SD* = 0.179) than low-frequency (*M* = 0.611, *SD* = 0.184) lists, *F*(1,48) = 28.855, *MSE* = 0.049, ${\eta}_p^2$ = 0.350, *p* < 0.001, BF_10_ = 812876. The main effect of block order was not significant, *F*(1,48) = 0.391, *MSE* = 0.419, ${\eta}_p^2$ = 0.008, *p* = 0.535, BF_01_ = 3.32. The main effect of position was significant, *F*(3.88,186.07) = 71.840, *MSE* = 0.037, ${\eta}_p^2$ = 0.599, *p* < 0.001, BF_10_ = 6.95 × 10^64^.

Unlike in Experiments 5 and 6, there was no interaction between block order and frequency, *F*(1,48) = 0.493, *MSE* = 0.049, ${\eta}_p^2$ = 0.010, *p* = 0.486, BF_01_ = 6.42. As can be seen in the right column of Fig. 4, a frequency effect was observed in both block orders. When the block of low-frequency lists occur first, performance is better for high-frequency lists (*M* = 0.675, *SD* = 0.207) than for low-frequency lists (*M* = 0.602, *SD* = 0.162), *t*(24) = 3.454, *d* = 0.691, *p* < 0.001, BF_10_ = 18.43. When the block of high-frequency lists occur first, performance is better for high-frequency lists (*M* = 0.717, *SD* = 0.147) than for low-frequency lists (*M* = 0.621, *SD* = 0.206), *t*(24) = 3.743, *d* = 0.749, *p* < 0.001, BF_10_ = 34.58.

The frequency by position interaction was significant, *F*(4.45,213.65) = 2.402, *MSE* = 0.021, ${\eta}_p^2$ = 0.048, *p* = 0.045, but the Bayesian analysis contradicted this result, BF_01_ = 9.30. The position by block order interaction was not significant, *F*(3.88,186.07) = 1.368, *MSE* = 0.037, ${\eta}_p^2$ = 0.028, *p* = 0.248, BF_01_ = 18.94. The three-way interaction was not significant, *F*(4.45,213.65) = 0.210, *MSE* = 0.021, ${\eta}_p^2$ = 0.004, *p* = 0.946, BF_01_ = 5.05 × 10^4^.

The main results of Experiment 7 are clear: When the subject does not know whether the test will be immediate serial recall or serial reconstruction of order, a frequency effect is observed regardless of whether the first block comprises low-frequency lists or high-frequency lists.

## General discussion

The item/order hypothesis developed out of the insight that item and order (or relational) information might trade-off in different tasks. It has been invoked to explain why some effects are observed only in within- and not in between-subject designs, and also to explain why some results with pure lists differ from those seen with mixed lists. Despite its simplicity, it has substantial scope. DeLosh and McDaniel (1996) applied the idea to frequency effects but focused on free recall where the role of order information may not be so clear. In contrast, the role of order information is more obvious in both immediate serial recall and serial reconstruction of order tasks. We derived predictions of the item/order hypothesis and searched the literature to assess its predictions; Table 1 summarizes this information. We then conducted experiments to fill in the empty cells in the table.

Experiment 1 replicated Quinlan et al. (2017) in supporting the prediction of a high-frequency advantage in serial reconstruction of order for pure lists when the lists are randomly ordered. The reason, according to the item/order hypothesis, is that pure lists should always result in a high frequency advantage because of the enhanced order information for the more common items. Experiment 2 confirmed the prediction of no frequency effect in serial reconstruction of order when the lists were changed from pure to mixed. The reason, according to the item/order hypothesis, is because alternating the two different kinds of items in the same lists reduces the order information for the high-frequency items while raising the order information for the low-frequency items. Presentation of the items at test offsets the item advantage for the low-frequency items. Together, this results in approximately equivalent performance.

Experiment 3 confirmed the prediction of a frequency effect when frequency is manipulated between subjects and the test is serial reconstruction of order. The reason, according to the item/order hypothesis, is that this experiment used pure lists, and pure lists should always result in a high frequency advantage for the reasons already noted.

Experiments 4 and 5 both examined pure lists but did so when the types of lists were blocked. Half the subjects received a block of high-frequency lists first followed by a block of low-frequency words second, and half received the reverse. The item/order hypothesis predicts a frequency effect because pure lists are used. Experiment 4 used immediate serial recall and supported the predictions. Experiment 5 used serial reconstruction of order but the prediction was supported only when the block of low-frequency lists came first; when the block of high-frequency lists came first, there was no frequency effect. Because of this unusual result, Experiment 6 was a partial replication, but despite the changes the same result obtained: A frequency effect was found when the low frequency block came first and was absent when the high frequency block came first.

Experiment 7 was a test of whether metacognitive factors might be causing the block order effect. The rationale was that if the type of test, immediate serial recall or serial reconstruction of order, was not known until after the list had been presented, the block order effect should be eliminated and a frequency effect observed for both block orders with serial reconstruction of order. The experiment confirmed the prediction.

The item/order hypothesis (DeLosh & McDaniel, 1996; Serra & Nairne, 1993) correctly predicts most of the cells in Table 1, with only two exceptions. First, it predicts that low-frequency words will be better recalled than high-frequency words on mixed lists with immediate serial recall. The reason is that in mixed lists, there should be roughly equal order information for all items because the presence of low-frequency items hurts order information for high-frequency items (relative to pure lists) but the presence of high-frequency items helps order information for low-frequency items (relative to pure lists). Because the low-frequency items retain their advantage for item information, the net result better recall of low-frequency items. However, there are at least three studies that show equivalent recall of high- and low-frequency items in immediate serial recall (Caplan et al., 2015; Hulme et al., 2003; Morin et al., 2006). We note that it was this prediction for mixed lists that also caused interpretive problems in the free recall literature, but whereas all three possible patterns were observed with free recall, only one pattern has been reported with serial recall.

Second, it predicts that high frequency words will be better recalled than low-frequency words in a blocked design with reconstruction of order tasks because by definition the lists are pure. In such lists, high-frequency words will have an advantage in order information, which is critical for these tests. However, Experiments 5 and 6 found that when the high-frequency block occurs first, there is no frequency effect: Performance is equivalent for the high- and low-frequency words. This is in stark contrast to the correct prediction for immediate serial recall, and for reconstruction of order when the first block contains low-frequency lists.

We will consider each of these predictions in turn. The first is more problematic for the item/order hypothesis than the second. It is difficult to modify the item/order hypothesis, and models that incorporate its ideas, to account for equivalent recall of high- and low-frequency words in mixed lists with immediate serial recall without losing the ability to account for other results. For example, the item/order account correctly predicts that when the test is serial reconstruction of order, performance will be equivalent for high and low-frequency items (see Experiment 2). The explanation is that presenting the items again at test can offset the item advantage for low-frequency words, resulting in equivalent performance.

One possibility is to assume that in mixed lists, the disruption of order information for high-frequency items caused by mixing high and low-frequency items might not be equivalent to the enhancement of order information for the low-frequency items. This could be the case because, for example, immediate serial recall might induce closer attention to order information than free recall, which was the basis for the original assumption (DeLosh & McDaniel, 1996). One consequence could be that the presence of additional order information due to task demands for low-frequency items combined with their advantage in item information renders them roughly equivalent to high-frequency items. This would enable the item/order hypothesis to account for the null effect of frequency in mixed lists with immediate serial recall. Note that this would not affect any of the other the predictions for immediate serial recall.

If this is the case, a key question is whether it affects the predictions for serial reconstruction of order. As currently posited, the relevant difference between immediate serial recall and serial reconstruction of order is that the low-frequency items lose some of their advantage in item information in the latter task. Whether the predictions change depends on the relative difference in the two types of information, but it is possible that the predictions would remain the same, both for mixed lists and for the other predictions for serial reconstruction of order. Ultimately, however, this is an empirical question and depends on careful comparisons of performance with mixed lists when four different tests — free recall, serial recall, serial reconstruction of order, and free reconstruction of order — are used.

The second notable prediction is less problematic if the cause of the block order effect is indeed a metacognitive one. The reason is that the item/order account does not include a metacognitive component, and therefore this is better classed as a phenomenon outside its purview. The block order effect is readily observed with other classes of stimuli; for example, Neath and Quinlan (2020) used a set of abstract and concrete words that were equated for frequency. When the abstract words occurred in the first block and the concrete words occurred in the second block, there was the usual concreteness effect. However, when the concrete words occurred in the first block and the abstract words in the second block, there was no difference on the reconstruction of order tests. As in Experiments 5 and 6, we tested both serial and free reconstruction of order, and open and closed sets. The pattern of results was identical. The same pattern occurs with manipulations of other variables such that the basic results may be stated as follows: When the test involves reconstruction of order, an effect of Variable X will be observed if the hard condition is in block 1 and the easy condition is in block 2. In contrast, if the easy condition is in block 1 and the hard condition is in block 2, there will be no effect.

## Summary

The item/order hypothesis is a general statement about how item and order information might trade-off in a variety of different paradigms. Despite its scope, it proposes a simple explanation for word frequency effects. However, the accuracy of those predictions has been difficult to assess in free recall because of the possible variation in the degree to which order information is required in that task. In contrast, we assessed its predictions using tests where the role of order information is less ambiguous. We have discussed two cases where the item/order hypothesis makes incorrect predictions. Strictly speaking, the hypothesis predicts a reverse frequency effect with mixed lists when tested by immediate serial recall. The extant data show no frequency effect, but reasonable modifications to the hypothesis have been described that can accommodate these findings. The other incorrect prediction was because the item/order account does not include a role for metacognitive processes. We have provided some initial evidence that the effect of word frequency in short-term memory tasks can itself be influenced by the amount of cognitive effort that the tasks demand.

## Appendix

**Table 2:** Stimuli used in Experiments 3–5

	High frequency				*t*	*p*	Low frequency			
	*M*	*SD*	Min	Max			*M*	*SD*	Min	Max
LgWF	3.32	0.43	2.71	4.75	**30.282**	**0.000**	1.91	0.50	0.30	2.71
LgCD	3.05	0.35	2.35	3.90	**31.061**	**0.000**	1.76	0.47	0.30	2.60
AoA	2	0	2	2	--	--	2	0	2	2
CNC	4.32	0.68	2.21	5	0.624	0.533	4.36	0.61	2.21	5
Celex	1.70	0.40	1.04	3.12	**33.859**	**0.000**	0.52	0.29	0.03	1.04
Orth	-0.19	0.72	-1.59	2.73	0.000	1.000	3.64	3.98	0	21
OrthZ	10.06	21.61	0	169.88	0.000	1.000	-0.19	0.72	-1.59	2.73
OrthF	3065.61	2365.10	88	13379	0.032	0.974	10.13	22.01	0	169.03
OLD	1.90	0.44	1	3.25	1.409	0.160	1.96	0.50	1	3.55
OLDF	7.48	0.72	4.76	9.32	1.017	0.310	7.40	0.77	5.48	9.10
PLD	1.73	0.54	1	3.75	1.133	0.258	1.80	0.64	1	4
PLDF	7.58	0.87	4.47	9.95	1.360	0.175	7.45	1.05	4.28	9.93
LgHAL	9.76	1.14	7.06	13.55	**22.648**	**0.000**	6.74	1.51	1.39	11.09
NLet	5.45	1.19	3	8	0.000	1.000	5.45	1.19	3	8
Nphon	4.42	1.10	2	7	0.091	0.928	4.43	1.09	2	8
Nsyll	1.56	0.57	1	3	0.356	0.722	1.54	0.55	1	3

Note: *LgWF* log frequency, *LgCD* log contextual diversity (from Brysbaert & New, 2009). *AOA* age of acquisition (from Brysbaert & Biemiller, 2017). *CNC* mean concreteness (from Brysbaert, Warriner, & Kuperman, 2014). *VM* mean valence, *AM* mean arousal, *DM* mean dominance (from Warriner et al., 2013). *CELEX* CELEX frequency, *Orth* number of orthographic neighbors, *OrthZ* z-transformed number of orthographic neighbors (see Storkel, 2004), *OrthF* frequency of orthographic neighbors (from Medler & Binder, 2005). *OLD* mean Levenshtein distance for the 20 closest orthographic neighbors; *OLDF* frequency of the 20 closest orthographic neighbors; *PLD* same as OLD except for phonological neighbors; *PLD* same as OLDF except for phonological neighbors; *LgHAL* log HAL frequency; *NLet* number of letters, *NPhon* number of phonemes, *NSyll* number of syllables (from Balota et al., 2007)

### High frequency

adult, alarm, anyone, babies, baby, balance, basket, bathroom, bedroom, belt, birth, blade, blanket, bomb, boss, bottom, bowl, bride, bridge, brush, bucket, bull, bush, business, cabin, chemical, chicken, chief, chin, church, circle, coast, collar, comfort, corner, crash, crime, cross, dark, desk, dining, dirty, double, draw, earth, elephant, empty, engine, express, face, families, farmer, finger, fish, fishing, floor, flower, football, fortune, freezing, fruit, garden, glass, gloves, goat, green, group, guard, handle, having, highway, hospital, hotel, human, hunt, hurry, husband, ice, inch, inside, iron, island, jacket, judge, jump, kids, kitchen, kitty, knife, ladies, lamb, language, laundry, lean, lips, lunch, magic, market, meeting, middle, milk, mirror, monster, moon, mother, motor, museum, myself, nails, navy, neck, nobody, noise, numbers, object, office, oil, once, open, opening, outside, package, painful, palace, paper, parade, people, permit, person, personal, pill, pipe, pistol, pitch, plant, pocket, pop, pound, pump, quarter, queen, rabbit, relax, rich, robin, roll, route, rubber, rug, running, salad, salt, scratch, scream, sign, single, skull, sleep, slide, smoke, snake, solid, son, song, southern, speed, stone, storm, student, study, sugar, summer, sunshine, supper, sweater, sweet, swim, swing, table, taste, team, throw, tissue, toast, toilet, tongue, touch, towel, track, trail, trip, troops, twins, valley, walker, warning, wash, wheel, whip, whistle, window, winter

### Low frequency

ache, acorn, airway, apron, arrow, bakery, bamboo, banjo, beagle, beak, beam, beetle, berry, blaze, blend, blinds, blossoms, blubber, bolts, bonnet, brace, brake, brat, broiler, brook, broom, buckle, buggy, bulb, burner, burp, buyer, carport, chalk, chimp, chirp, choke, cider, clap, claw, clip, coating, cobweb, coin, collie, colt, comma, cord, crank, crayon, croak, crumb, crunch, crust, crutch, dampness, dewdrop, dial, dimple, dipper, donkey, dryness, eardrum, eel, eyeglass, eyelid, farmyard, fawn, feelers, fern, ferry, fin, fishery, fizz, flap, flock, floppy, fluff, frosting, fudge, gallon, giggle, glare, grapes, grease, greens, grill, grind, grip, groan, growl, grunt, hairpin, hairy, heater, hitch, hobble, holdup, honeybee, honk, hoof, hothead, hound, hymn, igloo, ink, itch, kitten, kneel, knives, loaf, lung, mash, meanness, menu, mink, minnow, mitten, mixer, mop, neatness, noodle, orbit, ounce, outfield, overwork, oxcart, panda, parrot, paste, peas, pecan, pedal, peep, pickle, pigtail, plum, postage, quart, radish, raisin, ram, rattler, recount, redcoat, refill, reprint, reset, rhyme, roast, sandbag, saucer, seesaw, shaker, shopper, showman, sill, skate, skater, skunk, slacks, sled, slowness, smash, smog, snail, sneaker, snowfall, sock, spear, speller, spool, sprain, squeal, starch, steeple, sting, stool, sunburn, sunfish, suntan, swirl, tablet, talker, teapot, tickle, tuna, turnip, twig, uncut, uproar, usher, varnish, vest, vowel, waffle, wasp, wiper, woof, yarn, yawn, zebra

## Footnotes

1 The reason for the change in stimuli and list length is that Experiments 3–5 were conducted first. We then realized that two additional experiments would be informative. Experiment 1 allowed comparison of the online subjects to the university sample, and therefore the list length and stimuli needed to correspond to those in Quinlan et al. (2017). Experiment 2 was needed to complete the last cell in the table and keeping it similar to Experiment 1 allows for a direct comparison of pure and mixed lists. The order of reporting the experiments was changed to facilitate clarity of exposition.

2 The demographic data were inadvertently deleted.

3 The sample size increased from 40 to 50 due to an error setting up the study on Prolific.
